# Case Report: Aggressive neural crest tumors in a child with familial von Hippel Lindau syndrome associated with a germline *VHL* mutation (c.414A>G) and a novel *KIF1B* gene mutation

**DOI:** 10.3389/fendo.2023.1204793

**Published:** 2023-07-25

**Authors:** Lucie Landen, Anne De Leener, Manon Le Roux, Bénédicte Brichard, Selda Aydin, Dominique Maiter, Philippe A. Lysy

**Affiliations:** ^1^ Division of Pediatric Endocrinology, Specialized Pediatrics Service, Cliniques Universitaires Saint-Luc, Université catholique de Louvain (UCLouvain), Brussels, Belgium; ^2^ Division of Clinical Genetics, Cliniques Universitaires Saint-Luc, Université catholique de Louvain (UCLouvain), Brussels, Belgium; ^3^ Division of Pediatric Hematology and Oncology, Cliniques Universitaires Saint-Luc, Université catholique de Louvain (UCLouvain), Brussels, Belgium; ^4^ Division of Pathology, Cliniques Universitaires Saint-Luc, Université catholique de Louvain (UCLouvain), Brussels, Belgium; ^5^ Division of Endocrinology, Cliniques Universitaires Saint-Luc, Université catholique de Louvain (UCLouvain), Brussels, Belgium

**Keywords:** Von Hippel Lindau, pheochromocytoma, KIF1B gene, neural crest tumors, blood hypertension

## Abstract

**Introduction:**

Von Hippel Lindau (VHL) syndrome is caused by an autosomal dominant hereditary or sporadic germline mutation of the *VHL* gene with more than five hundred pathogenic mutations identified. Pheochromocytomas and rarely paragangliomas occur in 10-50% of patients with VHL syndrome usually around 30 years of age and exceptionally before the age of 10.

**Case presentation:**

We diagnosed a 9-year-old girl of normal appearance and severe refractory hypertension, with a norepinephrine-secreting pheochromocytoma related to VHL syndrome due to a known familial germline heterozygous mutation of *VHL* gene (c.414A>G), also present in three members of her family. At age 13, a pelvic tumor and a left adrenal pheochromocytoma that showed to be multi-metastatic to both lungs were discovered in the patient leading to left adrenalectomy and pelvic tumor resection. In addition to the germline *VHL* gene mutation, blood analysis using Next Generation Sequencing identified a novel heterozygous germline mutation of the *KIF1B* gene (c.3331_3332del; p.Asn1111Glnfs*21), which is only present in the girl and not the other family members. The patient is currently under steroid substitution therapy and leads a normal life.

**Discussion:**

This family is notable by the early age of onset of multiple neural crest tumors associated with a high propensity for malignancy and metastatic spread. Most reports in the literature associated the *VHL* mutation with a later onset in adulthood and a benign course, which contrast with our findings and question the role of this mutation in the phenotype expressed in this kindred. Also, the presence of concomitant mutations in two susceptibility genes for neural crest tumors poses the question of their respective roles in the development of tumors in this family. Our familial case description illustrates the potential for systematic use of targeted Next Generation Sequencing with multi-gene panels in patients with neural crest tumors to confirm the role of known susceptibility genes as well as identifying new ones, but also to contribute to comprehensive databases on gene variants and their phenotypic counterparts in this specific area of medicine.

## Introduction

Recent scientific evidence suggests that 30-40% of pheochromocytomas and paragangliomas (PPGL) result from germline mutations, making them the most frequently inherited neoplasias (1). Classical syndromic forms of these diseases include von Hippel Lindau (VHL) syndrome associated with *VHL gene* mutation, multiple endocrine neoplasia type 2 due to *RET gen*e mutation, neurofibromatosis type 1 due to *NF1 gene* mutation and familial PPGL syndromes due to *SDHx gene* mutations. The list of susceptibility genes associated with hereditary PPGL has been further extended over the last years and now also includes *TMEM127, MAX, MEN1, NF1, EGLN1/PHD2, EPAS1/HIF2A, FH* and *KIF1B* (2). Somatic mutations of the *RET, VHL, NF1, MAX, EPAS1/HIF2A* and *H-RAS* genes have also been described in sporadic pheochromocytomas.

VHL syndrome is caused by an autosomal dominant hereditary or sporadic germline mutation of the *VHL* gene mapped on the short arm of chromosome 3p25. More than five hundred pathogenic mutations of the gene have been identified including missense, deletion, nonsense, or frameshift mutations (3).

Clinical manifestations include central nervous system and retinal hemangioblastomas, clear cell renal cell carcinomas and cysts, pancreatic neuroendocrine tumors and cysts, endolymphatic sac tumors and epididymal cystadenomas. Pheochromocytomas and rarely paragangliomas occur in 10-50% of patients with VHL syndrome usually around 30 years of age and exceptionally before the age of 10, the youngest patient reported being 5 years old. VHL syndrome is the prevailing cause of pheochromocytomas in childhood accounting for 70% of cases. By comparison with deletions or loss of function variants, families with missense mutations are at much higher risk of developing pheochromocytoma, particularly codon 167 mutation carriers among which 80% of affected individuals present pheochromocytomas by the age of 50 (2).

Ten percent of pheochromocytomas and 15-35% of paragangliomas are malignant and metastatic disease is rare (2, 4). *SDHB gene* mutations are associated with the highest risk of malignancy (72%) and represent the most frequent cause of metastatic paragangliomas (2). Other genes much less frequently associated with malignancy include *VHL, NF1, SDHD* and *MAX*.

Herein we report a kindred of 4 patients presenting multiple neural crest tumors of childhood onset with a high propensity for malignancy and metastatic spread. Genotype analysis revealed a previously reported *VHL* gene germline mutation. In the youngest patient of this family a second, novel sporadic mutation in the *KIF1B* gene is reported.

## Patients and methods

A 9 year-old girl of normal appearance developed severe refractory hypertension and was diagnosed with a norepinephrine-secreting pheochromocytoma related to VHL syndrome (VHL) due to a known familial germline heterozygous mutation (class V variant) of the *VHL* gene (c.414A>G; p.Pro138Pro). Preoperative 24h urine measurements showed elevated norepinephrine (743 µg/24h; normal 10-100 µg/24h) and normetanephrine (5.0 mg/24h; normal 0.1-0.6 mg/24h). She underwent right adrenalectomy. Pathology failed to show criteria of malignancy apart from tumor necrosis (pheochromocytoma of adrenal gland scaled score (PASS) score: 2/20). Normal expression of succinate dehydrogenase A and B was found within the tumor. At age 13, while presenting with abdominal pain and sweating, the patient was diagnosed with a 40 mm left pelvic tumor located within the greater omentum (metastasis or paraganglioma) and with a 21 mm left adrenal pheochromocytoma that showed to be multi-metastatic to both lungs (pT1N0M1). She underwent a left adrenalectomy and pelvic tumor resection. In addition to the germline *VHL* gene mutation, blood analysis using Next Generation Sequencing of known susceptibility genes identified a novel heterozygous germline mutation of the *KIF1B* gene (c.3331_3332del; p.Asn1111Glnfs*21) (variant allele frequency of 50.6%). According to the algorithms of the American College of Medical Genetics and Genomic (ACMG) and the Association for Molecular Pathology (5), the novel variant is considered as “likely pathogenic” (class IV variant). Genome sequencing of exon 30 of the *KIF1B* gene on a maternal blood sample revealed no mutation. The girl’s father has yet to be tested for *KIF1B* gene mutation. We believe the KIF1B mutation found in our 9-year-old patient corresponds to a germline origin as the genetic determination was obtained from a blood and not tissue sample. The heterozygous presence of the mutation was confirmed using Sanger sequencing (no peak changes).

**Figure 1:**
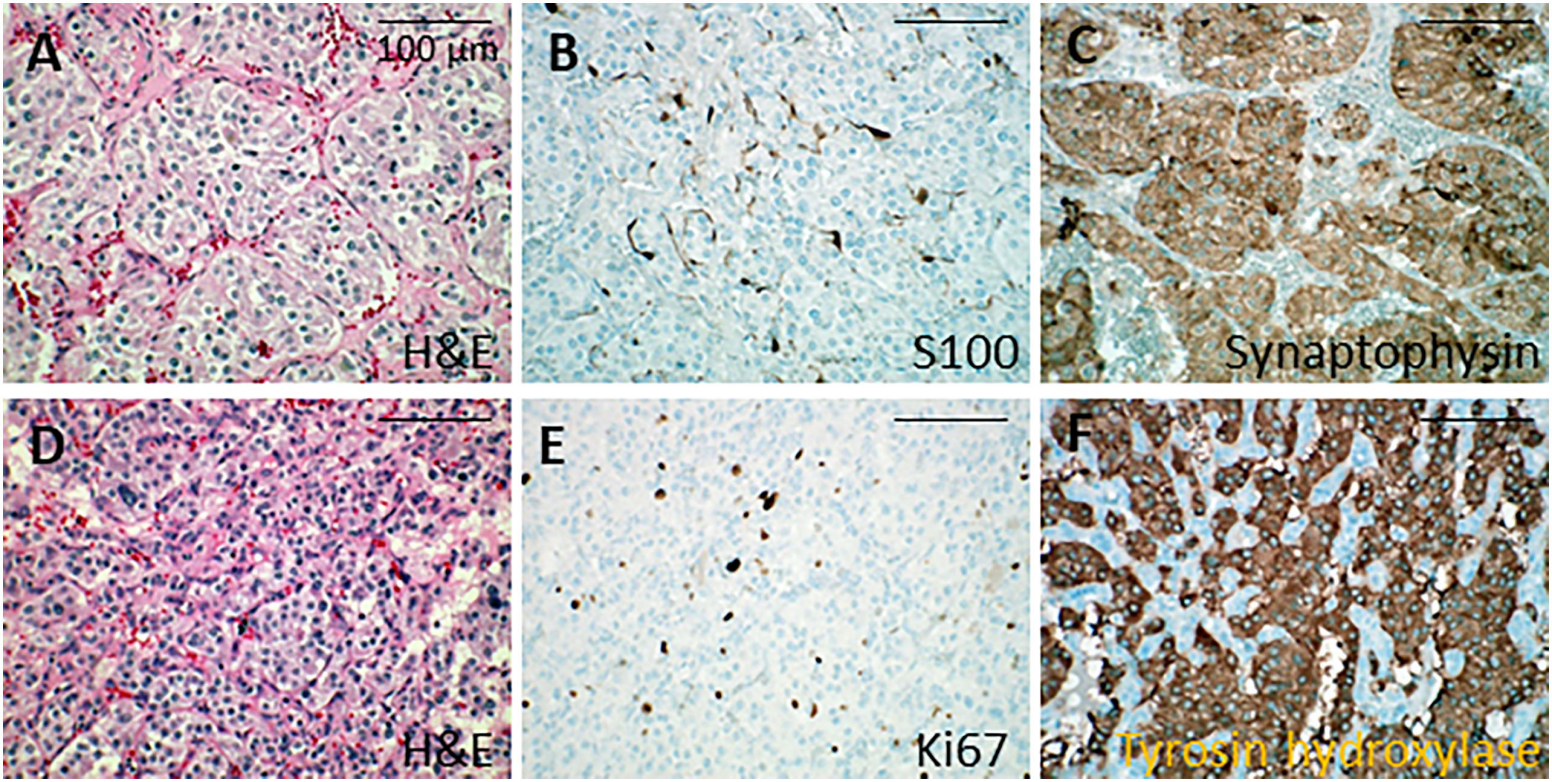
Histological analysis of adrenal tumors. **(A-C)** Microphotographies of the first tumor in the right adrenal gland. **(A)** Nest arrangement of neoplastic cells showing mild atypias (hematoxylin and eosin). **(B)** S100 protein immunolabeling in the sustentacular cells. **(C)** Synaptophysin immunolabeling in the cytoplasm of neoplastic cells. **(D-F)** Microphotographies of the second tumor in the left adrenal gland. **(D)** Neoplastic cells with moderate atypias forming trabeculae (hematoxylin and eosin). **(E)** Ki67 immunolabeling in the nucleus of the neoplastic cells, showing moderate proliferation. **(F)** Tyrosine hydroxylase immunolabeling in the cytoplasm of neoplastic cells. Bars, 100 µm. H&E, hematoxylin and eosin.

The adrenal tumor consisted of a 21 mm nodule with histological features similar to the previously resected pheochromocytoma except for invasion of an adrenal vein by tumor cells (PASS score: 1/20). The pelvic tumor specimen revealed a 40 mm tumor and 6 mm satellite nodule, both surrounded by a fibrous capsule. Tumor cells were similar to those observed in the pheochromocytoma but exhibited hyperchromatic nuclei and nuclear pleiomorphism (**Figure 1**). Tumor cells infiltrated the tumor capsule and spread focally into the surrounding adipose tissue. Lymphovascular permeations were present as well as a microscopic tumor deposit on the peritoneum. Immunohistochemistry showed normal expression of tyrosine hydroxylase as well as succinyl dehydrogenase A and B (**Figure 1**). The KI67 index was 15%, focally attaining 20%. Sanger sequencing on the adrenal tumor revealed the same heterozygous germline mutations of the *VHL* and *KIF1B* genes described above.

Resection of the pelvic tumor was incomplete (R1) according to pathological findings and a post-operative MIBG scan indeed showed moderate residual fixation in the left iliac fossa. Further treatment consisted of two courses of metabolic radiotherapy using I^131^-radiolabeled MIBG at the dose of 150 mCi in September 2020 and 202 mCi in February 2021, yielding a partial response of the lung metastases. At 2 years follow-up there are no clinical, biological, or radiological signs of abdominal neuroendocrine tumor recurrence. Ser

um chromogranin A and urinary catecholamines are within normal range. Chest CT scan shows mild regression in size of the pulmonary metastases. The patient is currently under steroid substitution therapy and leads a normal life. She exhibits no signs of other anomalies linked to VHL syndrome (retinal/cerebral/spinal hemangiomas, renal tumors/cysts, pancreatic neuroendocrine tumors/cysts).

The proband of the family was the patient’s mother who was diagnosed at 12 years of age with VHL syndrome (*VHL* germline mutation (c.414A>G; p.Pro138Pro)), when she had resection of bilateral pheochromocytomas. Other manifestations of VHL syndrome included millimetric hemangioblastomas in the right retina, cervical, dorsal and lumbar spines, a 2 cm pancreatic tail cystadenoma and a 9 mm right renal cyst of benign aspect. A hematocele was also noted, which, in the context of VHL syndrome might be a papillary cystadenoma of the broad ligament having bled. She has a history of congenital aortic valve stenosis and interventricular septal defect operated at age 17. Furthermore, she had resection of a malignant schwannoma of the right shoulder at 21 and underwent total thyroidectomy for papillary cancer at age 40. She is alive at age 48.

**Figure 2:**
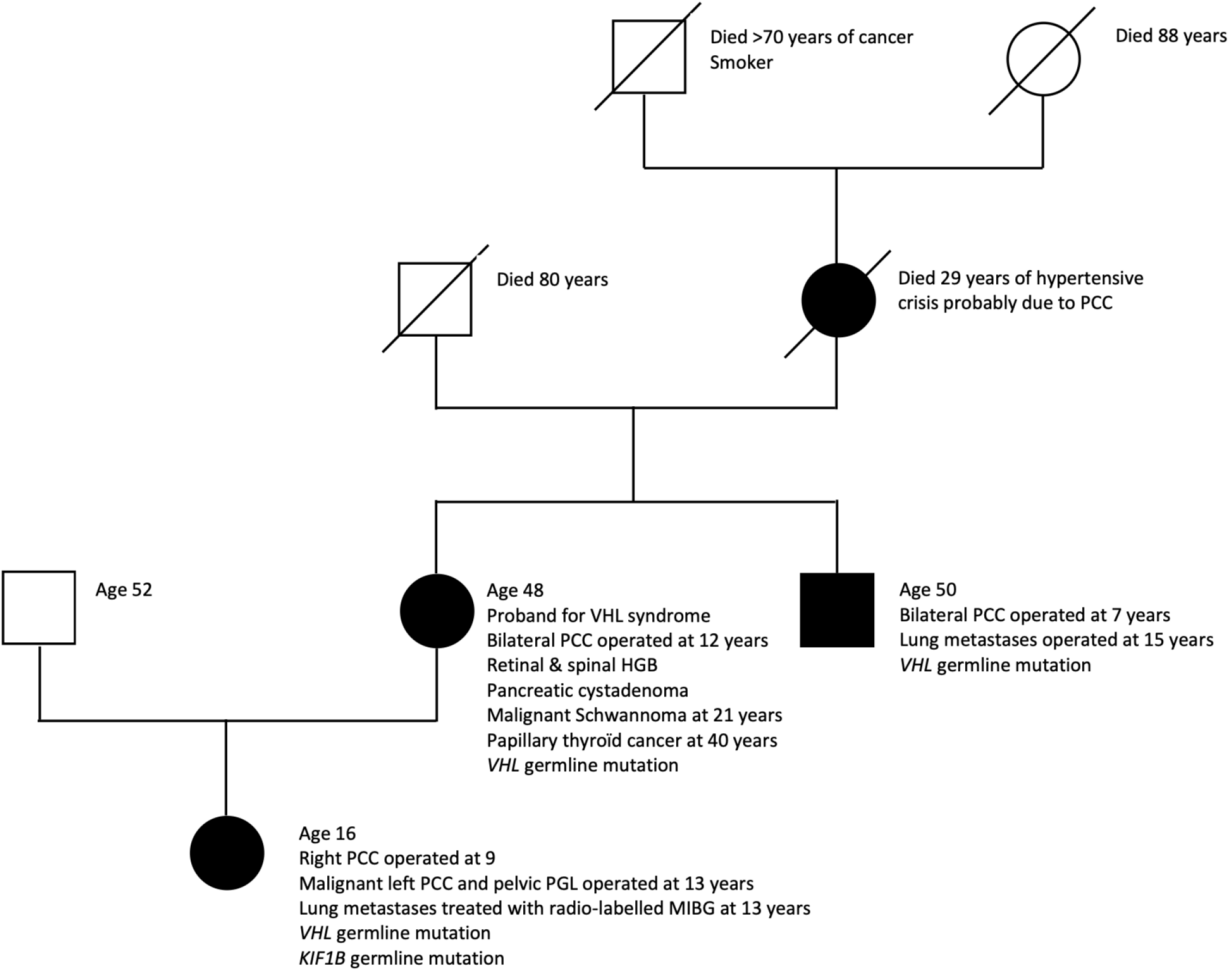
Pedigree of family with early-onset aggressive neural crest tumors. PCC, pheochromocytoma; PGL, paraganglioma; HGB, hemangioblastoma.

The girl’s maternal uncle was diagnosed with VHL syndrome secondary to the same VHL gene mutation and underwent bilateral adrenalectomy for pheochromocytoma at age 7 followed by resection of lung and abdominal metastases at age 15. He declined further follow-up and is alive at age 50. **Figure 2** resumes the family pedigree.

The girl’s grandmother died at age 29 of a hypertensive crisis due to a right-sided pheochromocytoma diagnosed at autopsy. She had previously undergone left adrenalectomy for pheochromocytoma.

The Human Gene Mutation Database (HGMD® Professional 2022.2) and the human variant database ClinVar™ of the National Institutes of Health were searched for variants of *VHL* and *KIF1B* genes and a literature search was performed on the National Library of Medicine PubMed® database using the search terms pheochromocytoma, von Hippel Lindau, *VHL* gene, *KIF* gene.

## Discussion

The *VHL* gene codes for a tumor suppressor protein (pVHL) that regulates the cellular oxygen-sensing pathway through modulation of hypoxia-inducible factor alpha (HIFalpha) activity. VHL protein also plays a role in many other cellular processes including cilia formation, cytokine signaling, regulation of senescence, and formation of the extracellular matrix. In hypoxic conditions HIFalpha and HIFbeta interact, inducing the transcription of hypoxia-inducible genes. This enhances the expression of angiogenic growth and mitogenic factors (vascular endothelial growth factor, platelet-derived growth factor, erythropoietin, and transforming growth factor alpha) which contribute to tumor formation (2). By reproducing pseudohypoxic states, mutations in *VHL, SDHx* and *HIF2A* genes induce the development of multiple microscopic foci of neoplastic precursor lesions, a minority of which seem to develop into overt tumors including pheochromocytomas and paragangliomas (2, 3). In this context, it may be of significance that the proband in this kindred had a history of congenital aortic valve stenosis and interventricular septal defect operated at age 17, a condition which may have been responsible for chronic hypoxia, accentuating HIF activity. The proband also developed a malignant shoulder schwannoma at 21 and papillary thyroid cancer at 40 years of age. *NF1* and *NF2* genes are pathogenic for schwannoma and *BRAF*, *RAS* and *RET* mutations for papillary thyroid carcinoma. However, involvement of the *VHL* gene mutation in the genesis of these tumors is not established.

In so-called synonymous or “silent” mutations the replacement of a nucleotide by another does not alter the sequence of amino acids. However, the mutation may still have consequences on protein synthesis and function. In the case of the *VHL* mutation c.414A>G; p.Pro138Pro, evidence suggests that this results in altered pre-mRNA splicing. The *VHL* gene comprises 3 exons which code for two protein-coding transcripts. The long isoform comprises exons 1,2 and 3 whereas the short isoform lacks exon 2. The former codes for two proteins containing 213 and 160 amino acids, both of which are functional tumor suppressors. The isoform lacking exon 2 codes for a 172 amino acid protein that is believed to lack tumor suppressor properties (6). Evidence suggests that although tumors arise predominantly from missense mutations, splice-altering mutations can also lead to tumor formation (6–8). For disease to occur it is believed that the second wild-type allele must be inactivated through somatic mutation, deletion, or through hypermethylation of its promoter (“two-hit” model). This loss of heterozygosity through inactivation of the wild-type allele is probably determinant for tumor development (7).

In the human variant database ClinVar™ of the National Institutes of Health we found three reports of a similar mutation of the *VHL* gene (c.414A>G; p.Pro138Pro) involving 29 affected individuals from 8 independent families (**Table 1**). Another 15-year-old patient with bilateral pheochromocytomas is reported in a series of 314 Chinese patients (4).

**Table 1:** Published kindreds with neural crest tumors and *VHL* c.414A>G (p.Pro138Pro) mutation.

Author (year)	Lenglet 2018	Lenglet 2018	Flores 2019	Flores 2019	Flores 2019	Flores 2019	Flores 2019	Liu 2020	Ma 2020	Landen 2022
**Kindred size (n)**	3	4	7	5	3	3	1	3	1	4
**Age of proband at onset (years)**	NA	NA	32	53	31	20	47	47	15	12
**Age range at diagnosis (years)**	NA	NA	12-73	29-64	31-50	20-27	47	6-47	15	7,9,12,<30
**Gender F:M**	1:2	3:1	2:5	1:4	0:3	0:3	1:0	2:1	0:1	3:1
**Phenotype**	PCC 3 HGB 2 RCC 1	PCC 4	PCC 7 HGB 1/7	PCC 5 PGL 1/5 HGB 1/5	PCC 3	PCC 3 HGB 1/3	PCC PGL	PCC 1/3 HGB 3/3	PCC	PCC 4/4 PGL ¼ HGB 1/4
**Presentation**	NA	PCC	PCC	PCC	PCC	PCC	PCC	HGB+PCC	NA	PCC
**Multiple PCC**	NA	NA	0/7	3/5	0/3	2/3	1/1	0/3	1/1	4/4
**Malignancy**	renal	NA	No	Lung	No	No	No	No	NA	Lung 2/4
**Other VHL manifestation**	PGL	No	Spinal HGB	Spinal HGB	No	Spinal HGB	No	Spinal, retinal HGBs	NA	Spinal, retinal HGBs
**VHL subtype**	NA	NA	2A	2A	2C	2A	2C	2A	NA	2A+2C
**Other tumors**	NA	No	Thyroid nodule, bladder carcinoma	No	No	Parathyroid nodule, esophageal carcinoma	no	no	NA	Schwannoma, thyroid carcinoma (papillary)
**Follow-up**	NA	NA	30 years	18 years	10 mos	25 years	20 years	10 years	NA	

PCC, pheochromocytoma; PGL, paraganglioma; HGB, hemangioblastoma; NA, information unavailable.

According to criteria of the ACMG this mutation is classified as pathogenic (class V) and responsible for VHL syndrome. In these previously reported cases, the average age at diagnosis was 35 years (12-64 years) with a male predominance of 2:1. Pheochromocytomas, often multiple, constituted the initial manifestation of the disease in all patients but were malignant in one patient only. By comparison, the kindred reported herein were diagnosed in childhood and were predominantly female. The present family is remarkable by the early age of onset of pheochromocytomas (7,9 and 12 years), the fact that all individuals developed bilateral tumors, the high rate of malignancy (3/4) and of lung metastases (2/4).

If indeed the *VHL* mutation is responsible for the phenotype expressed in this kindred, this contrasts with most reports in the literature in which tumors occur later in adulthood and follow a benign course. Retinal and central nervous system hemangioblastomas are usually the first manifestation of VHL syndrome, affecting 70% of individuals at the age of 25. At 16 years of age our patient has not developed other classical characteristics of VHL syndrome, which is consistent with VHL syndrome type 2C. The catecholamine secretion profile of our patient was consistent with VHL disease in which pheochromocytomas secrete mostly norepinephrine due to the usually low or absent expression of phenylethanolamine N-methyltransferase (2).

The *KIF1B* gene (kinesin family member 1b) located on chromosome 1p36.22 codes for two protein isoforms pKIF1Bα and pKIF1BBβ, involved in axonal growth, myelination, and transport of synaptic vesicles and mitochondria (9).

Rare germline mutations of this gene have so far been mostly associated with Charcot-Marie-Tooth disease type 2A, a sensitive and motor neuropathy (10). KIF1Bβ protein is required for induction of apoptosis in particular cells types, through upregulation of prolyl hydroxylase Egln3/PHD3. Loss of function of the gene allows sympathetic precursor cells to escape cell death (1, 11). Various mechanisms have been hypothesized to explain pathogenicity including haploinsufficiency of *KIF1B*, epigenetic silencing of wild-type allele in the tumor (10, 11), and loss of heterozygosity (11).

There are only six reports (**Table 2**) (1, 4, 10, 12–14) of patients with neural crest tumors harboring *KIF1B* germline mutations. All patients were female, with an average age of 38 years (22-54 years) and no reports of childhood onset. Tumors were located in the adrenals except for one patient with a bladder paraganglioma. The secreted hormones were norepinephrine (5/6 patients) and epinephrine (2/6 patients). No patient had a family history of neural crest tumors except for the first case published in 2008 but the authors concluded in a later report that the family phenotype probably resulted from a *MAX gene* germline mutation and not the *KIF1B* mutation (10) The absence of familial clustering in the patients with *KIF* mutations could be explained by a sporadic germline mutation that could affect future offspring. It could also be due to a low penetrance of the disease and require further follow-up of unaffected family members. In the family followed by our team the VHL germline mutation is an established susceptibility gene predisposing to neural crest tumors and only one member harbors a second mutation involving the *KIF* gene, of unknown significance according to ACMG criteria. The gene mutation of the *KIF1B gene* (c.3331_3332del; p.Asn1111Glnfs*21) in our patient has not been reported previously.

**Table 2:** Patients with neural crest tumors and KIF1B mutations.

Reference Year	(4) 2020	(12) 2020	(11) 2020	(13) 2014	(9) 2008	(1) 2021	Landen 2022
**Age (years)**	36	48	46	54	22	24	9
**Gender**	F	F	F	F	F	F	F
**Tumor location**	Uni-PCC	PGL	Uni-PCC	Uni-PCC	Bi-PCC	Uni-PCC	Bi-PCC + PGL
**Familial**	No	No	No	NA	Yes	No	Yes
**Metastases**	Yes	No	Lymph node?	No	No	No	Lung
**Nmetanephrine**	NA	NA	elevated	NA	NA	elevated	elevated
**Metanephrine**	NA	NA	elevated	NA	NA	normal	normal
**Nepinephrine**	elevated	NA	NA	elevated	elevated	elevated	elevated
**Epinephrine**	elevated	NA	NA	normal	normal	normal	normal
**Dopamine**	normal	NA	NA	NA	NA	normal	normal
**Other tumors in kindred**	no	no	no	Endometrial carcinoma	Neuroblastoma lymph node leiomyosarcoma lung cancer	no	Schwannoma Thyroïd cancer (papillary)
**Mutation**	*KIF* germline	*KIF* germline	*KIF* germline	*KIF* germline	*KIF* germline *MAX* germline	*KIF* germline	*KIF* sporadic *VHL* germline

PCC, pheochromocytoma; PGL, paraganglioma.

It is likely that the *VHL* mutation played a predominant role in the early and aggressive development of tumors in this patient. However, a cumulative or potentiating role of this second *KIF* mutation in the genesis of tumors in the youngest patient cannot be excluded. It can be hypothesized that both mutations act synergistically, the *VHL* mutation inducing decreased tumor suppression through the oxygen-sensing pathway and the *KIF* mutation being responsible for reduced apoptosis through kinase-signaling. The report of other tumors in patients with *KIF* mutations (endometrial carcinoma, neuroblastoma, ganglioneuroma or lung adenocarcinoma) may indicate susceptibility to tumors derived from other embryonic cell lineages.

The presence of concomitant mutations in two susceptibility genes for neural crest tumors poses the question of their respective roles in the development of tumors in this family. Because of complexity owing to its large size, mutations of the *KIF1B* gene have not been routinely investigated. In the future the systematic use of targeted Next Generation Sequencing with multi-gene panels in patients with neural crest tumors will confirm the role of known susceptibility genes as well as identify new ones, contributing to comprehensive databases on gene variants and their phenotypic counterparts. Neural crest tumors stored in tissue banks can also be investigated a posteriori using new multi-gene panels and thus contribute to the identification of susceptibility genes. The present report adds weight to the role of the *VHL* gene variant c.414A>G and possibly of the *KIF1B* gene variant c.3331_3332del in the genesis and development of neural crest tumors.

## Data availability statement

The original contributions presented in the study are included in the article/supplementary materials. Further inquiries can be directed to the corresponding author.

## Ethics statement

Written informed consent was obtained from the individual(s), and minor(s)’ legal guardian/next of kin, for the publication of any potentially identifiable images or data included in this article.

## Author contributions

LL wrote the manuscript. AL performed the genetic studies, revised the study methods and edited the manuscript. BB performed clinical follow-up, designed the study and edited the manuscript; SA performed the pathology studies and edited the manuscript. DM performed clinical follow-up, revised the study and edited the manuscript. PL performed clinical follow-up, designed the study and wrote the manuscript. All authors contributed to the article and approved the submitted version.
